# Collaborative Attitudes and Their Relationship with Empathy and Personality Traits among Medical Residents: A Correlational Study

**DOI:** 10.1192/j.eurpsy.2026.12023

**Published:** 2026-07-31

**Authors:** A. Alcorta-Garza, O. Vidal-Gutiérrez, J. A. Martínez-Moyano, C. B. González-Alcorta, F. Alcorta-Núñez, M. L. Garza-García, P. A. López-Sierra, I. L. Galaviz-Reynoso, A. M. Cortés-Almazán, C. A. Martínez-Roque, J. F. González-Guerrero

**Affiliations:** 1 University Center Against Cancer; 2 Oncology Service, UANL, Monterrey, Mexico

## Abstract

**Introduction:**

Few studies have examined attitudes toward collaborative work among medical residents.

**Objectives:**

We evaluated collaborative attitudes and examined their relationships with empathy, personality traits, covert narcissism, and affective–cognitive dysregulation, among medical residents.

**Methods:**

All medical residents at a public tertiary care teaching hospital were invited to participate in the survey; 213 completed the anonymous online questionnaire (77% response rate). Spearman correlation coefficients were calculated between scores on the Jefferson Scale of Physician Empathy for Physicians and Health Professionals, the Hypersensitive Narcissism Scale, the Zuckerman–Kuhlman Personality Questionnaire, the Affective–Cognitive Dysregulation Scale, and the Jefferson Scale of Attitudes Toward Interprofessional Collaboration (JeffSATIC).

**Results:**

The mean overall collaborative attitude score was 118.8 ± 15.4 (range: 20 -140). Empathy was positively correlated with working relationships (ρ = 0.408) and accountability (ρ = 0.372). In contrast, aggression–hostility (ρ = –0.156 and –0.177, respectively) and affective–cognitive dysregulation (ρ = –0.244 and –0.259, respectively) were negatively correlated with these outcomes. Covert narcissism, impulsive sensation seeking, and neuroticism–anxiety were negatively associated with accountability but showed no relationship with working relationships.

**Conclusions:**

Some factors were associated with both JeffSATIC dimensions —working relationships and accountability —while others were linked only to one. The findings suggest that teamwork is not a single construct and that enhancing accountability may require addressing specific personality traits.

**Disclosure of Interest:**

None Declared

